# Safety and clinical efficacy of linezolid in children: a systematic review and meta-analysis

**DOI:** 10.1007/s12519-022-00650-1

**Published:** 2022-12-23

**Authors:** Yi Shi, Hai-Lan Wu, Yu-Hang Wu, Shuang Li, Li-Ya Zhang, Shan-Shan Xu, He-Yu Huang, Chun-Hong Zhang, Xu-Ben Yu, Kang Cai, Jing Zhang, Li-Su Huang

**Affiliations:** 1 Department of Infectious Disease, Xinhua Children’s Hospital, Xinhua Hospital, Shanghai Jiao Tong University School of Medicine, Shanghai, 200092 China; 2 National Clinical Research Center for Child Health, Children’s Hospital, Zhejiang University School of Medicine, Hangzhou, 310052 China; 3 Institute of Antibiotics, Huashan Hospital, Fudan University, Shanghai, 200040 China; 4 Department of Pharmacy, the First Affiliated Hospital of Wenzhou Medical University, Wenzhou, 325000 China

**Keywords:** Children, Efficacy, Linezolid, Meta-analysis, Safety

## Abstract

**Background:**

We aimed to evaluate the tolerability and efficacy of linezolid in children for treating suspected and diagnosed Gram-positive bacterial infections.

**Methods:**

A systematic literature search was conducted up to April 23, 2021, using linezolid and its synonyms as search terms. Two reviewers independently identified and extracted relevant randomized controlled trials and prospective cohort studies. The extracted studies were included in a single-rate meta-analysis of adverse events and clinical outcomes using random-effects models.

**Results:**

A total of 1082 articles were identified, and nine studies involving 758 children were included in the meta-analysis. The overall proportion of adverse events was 8.91% [95% confidence interval (CI) = 1.64%–36.52%], with diarrhea (2.24%), vomiting (2.05%), and rash (1.72%) being the most common. The incidences of thrombocytopenia and anemia were 0.68% and 0.16%, respectively. Some specific adverse events, including rash and gastrointestinal events, were more frequent in the oral administration subgroup. In terms of efficacy, the overall proportion of clinical improvement was 88.80% (95% CI = 81.31%–93.52%). Children with a history of specific bacteriological diagnosis or concomitant antibiotic therapy had a 1.13-fold higher clinical improvement than children without such histories. The proportion of microbial eradication was 92.68% (95% CI = 84.66%–96.68%). The proportion of all-cause mortality was 0.16% (95% CI = 0.00%–7.75%).

**Conclusions:**

Linezolid was well-tolerated in pediatric patients and was associated with a low frequency of adverse events, such as anemia, thrombocytopenia, and neutropenia. Moreover, linezolid was effective in children with diagnosed and suspected Gram-positive infections.

**Supplementary Information:**

The online version contains supplementary material available at 10.1007/s12519-022-00650-1.

## Introduction

Methicillin-resistant *Staphylococcus aureus* (MRSA) infections have decreased overall but have increased in children [1]. Linezolid is a synthetic oxazolidinone antibiotic that was approved for children in 2002 by the U.S. Food and Drug Administration to treat pneumonia caused by Gram-positive cocci, skin or skin soft tissue infections (SSSIs), and infections caused by vancomycin-resistant *Enterococcus faecium* (VRE). Linezolid is recommended as an alternative to vancomycin, a first-line drug, for treating resistant Gram-positive bacterial infections, such as MRSA osteoarthritis. With the increased use of anti-MRSA agents, the trend of vancomycin and linezolid use demonstrated a significant increase from 2006 to 2015 in Japan [2]. However, the safety and efficacy of linezolid in children remain understudied.

In terms of safety, the incidence of major linezolid-related adverse events in children has been reported inconsistently. For example, the most commonly reported general adverse events include diarrhea (7.8%–16.8%) and vomiting (2.9%–11.9%) [3]. Myelosuppression, which is a serious adverse event, occurs in 0%–24% of children and leads to the discontinuation of linezolid in some cases [4]. This range is quite broad, making it difficult for clinicians to determine whether a child is at a high risk for a particular adverse event and requires careful monitoring or discontinuation of treatment. Second, the judgment and treatment of adverse events in children are based on adults, but the difference between children and adults makes it flawed in clinical practice. For example, the most common types of myelosuppression in adults are thrombocytopenia and leukopenia, but manifestations other than thrombocytopenia in children are rarely reported. First-line clinical trials need to be summarized to present the actual situation in children. In addition, some adult studies have attributed more adverse events to oral administration, such as more gastrointestinal disturbances in the treatment of SSSI [5, 6]. However, the effects of different routes of administration in children have not been studied.

Linezolid has high tissue penetration and is recommended for the treatment of Gram-positive infections, such as pneumonia in children. Linezolid has a high clinical cure rate in treating SSSIs, bacteremia, and pneumonia in children, but there is still uncertainty. The cure rate was found to be only 69% in a clinical study of treating acute otitis media in children [7], while in another clinical study, it achieved 100% [8]. Considering the antibacterial spectrum of linezolid, we speculate that the etiological diagnosis may have a great impact on the efficacy.

Based on the above analysis, there is still much variability regarding the safety and efficacy of linezolid in children. As of November 2021, there have been two meta-analyses on the tolerability or efficacy of linezolid for treating nontuberculous infections in children. One of them was published in 2014; however, much research has been done since then [9]. The other meta-analysis only addressed myelosuppression and included retrospective studies [4]. Thus, we conducted a meta-analysis of prospective studies to clarify the adverse events and effectiveness of linezolid in children. Considering the heterogeneity of the studies, subgroup analyses were performed based on the route of administration, race, etc.

## Methods

### Search strategies

A search of PubMed and Embase was performed (up to April 23, 2021). Search terms included “linezolid” and its synonyms “linezolide, zyvox”. Only articles published in English were considered. The study population was children under 18 years of age. The specific search in PubMed included “linezolid” [MeSH terms] OR “linezolid” [title/abstract] OR “linezolid” [text word]. The search term in Embase included (linezolid:ti, ab, kw OR “linezolid”/exp/mj) AND ([newborn]/lim OR [infant]/lim OR [child]/lim OR [preschool]/lim OR [school]/lim OR [adolescent]/lim) AND [humans]/lim AND [English]/lim.

### Selection criteria

Articles were selected using EndNote software (Version X9, USA), and duplicate articles were deleted. Two reviewers independently examined the title and abstract. The original articles were searched as appropriate. Our inclusion criteria were as follows: studies of linezolid in children under 18 years of age, prospective studies, and studies of the efficacy or associated adverse events of linezolid. We excluded retrospective studies, reviews or meta-analyses, studies with sample sizes of less than five participants, case reports and case series, cell research studies, studies related to *Mycobacterium tuberculosis* infection, and nonclinical studies. The quality of the article was assessed using the Agency for Healthcare Research and Quality (AHRQ) guidelines. All studies were combined for analysis if subgroup analysis stratified by study design showed no significant difference between randomized controlled trials (RCTs) and other prospective studies.

### Data extraction

The following data were extracted: author, year of publication, study design, sample size, characteristics of patients (age, race, sex, weight and height), underlying disease, site of infection, pathogen and culture, concentration monitoring of linezolid, concomitant drugs (antibiotics, such as aztreonam against Gram-negative bacteria, and other special treatment drugs, such as hormones), application of linezolid (dosage, route of administration, and treatment course), clinical improvement (including clinical cure), microbiological efficacy (pathogen eradication), all-cause mortality, and adverse events (rash, nausea, vomiting, diarrhea, loose stools, abdominal pain, discoloration of tongue, thrombocytosis, acute pancreatitis, renal damage, hepatic damage, leukopenia, anemia, thrombocytopenia, lactic acid rise and neutropenia). Important missing data were requested from the author by e-mail. Dosage data were not found in the original article and supplemental materials, and there was no reply after writing to the author. Thus, the recommended dosage for children of this age was applied for analysis.

### Statistical analysis

The outcome indicators are presented as numbers and percentages. The outcomes were analyzed via single-rate meta-analysis with the random intercept logistic regression model. The forest plot was drawn according to the obtained results. Subgroup analysis was performed in groups with no less than two studies after stratification using the inverse variance method. The group analysis was based on categorical variables. Studies were stratified into two or three groups according to the following characteristics: pathogen (patients diagnosed with Gram-positive bacterial infections vs. patients with presumed or unclear pathogenic diagnosis), daily dose (patients treated with linezolid > 30 mg/kg/day vs. < 30 mg/kg/day), route [oral and intravenous changed to oral (sequential therapy) vs. intravenous], adjustment of dosage (dosage of linezolid adjusted as needed vs. no adjustment of dosage considered), duration of treatment (more than 14 days vs. less than 14 days), combination of antibiotics (concomitant antibiotics such as aztreonam used as needed vs. no concomitant antibiotics), combination of other drugs (concomitant other drugs such as glucocorticoid used as required vs. no concomitant other drugs), race (white vs. other races accounting for the largest proportion of patients), and sample size (less than 30 children vs. more than 30 children). To assess the frequency of adverse events, incidences between 1% and 10% were considered common adverse events. Rare events were defined as events with an incidence of < 1%. The meta-analysis was performed by R software (4.1.1, New Zealand), and some charts were generated in Excel and GraphPad Prism (7.00, USA). In the meta-analysis, random-effects models were selected.

## Results

### Main characteristics of the pooled trials

**Fig. 1:**
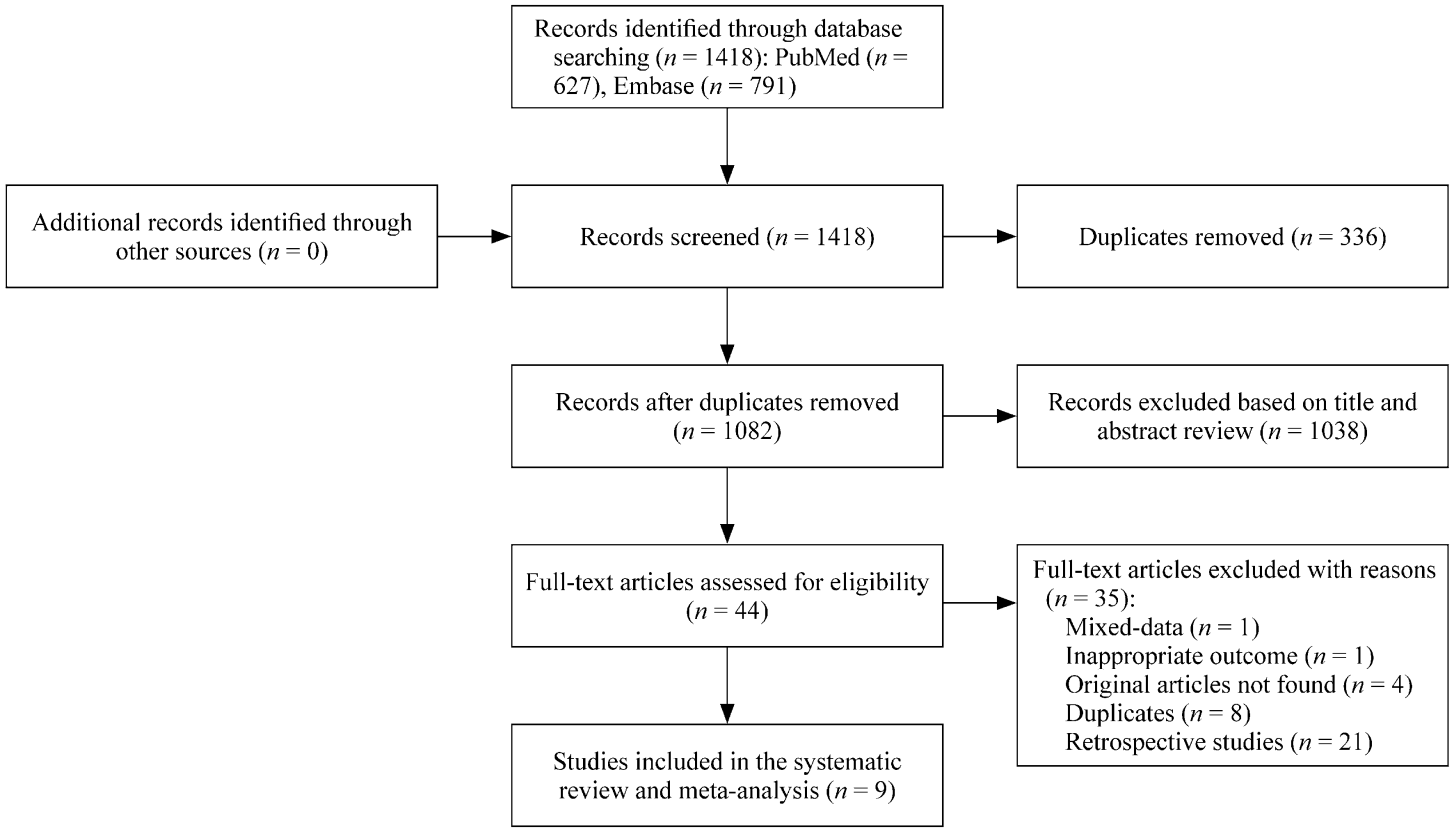
Flowchart of this study

The search results are shown as a flowchart in Fig. 1. A total of 1082 articles were identified. Forty-four studies were screened out according to the title and abstract and were assessed for eligibility. Thirty-five articles were removed. Of these, 21 were retrospective studies, eight were secondary analyses of primary data, four were conference abstracts for which we wrote to the authors requesting articles and did not receive a response, one had inappropriate outcomes, and one did not separate the data from adults and children. Finally, nine studies were included in this meta-analysis. Two of them were RCTs [10, 11], and the rest were uncontrolled clinical trials [8, 12–17]. The range for the quality score of the included trials was 5–10 (AHRQ). The safety and efficacy of linezolid in children were analyzed according to the information extracted from each study.

### Adverse events

Adverse events were assessed in 758 patients from nine studies [8, 10–17]. Overall, the proportion of adverse events after linezolid therapy was 8.91% [95% confidence interval (CI) = 1.64%–36.52%] (Table 1). Regarding specific adverse events, diarrhea was the most common (2.24%, 95% CI = 0.43%–10.81%), while anemia (0.16%, 95% CI = 0.00%–5.78%) was the least common. Other common specific adverse events included vomiting (2.05%, 95% CI = 0.99%–4.17%) and rash (1.72%, 95% CI = 1.00%–2.93%). Additional uncommon specific adverse events included nausea (0.96%), loose stools (0.3%), abdominal pain (0.23%), hepatic injury (0.51%), thrombocytopenia (0.68%), and neutropenia (0.21%). The following specific adverse events were excluded, since they were recorded in less than two studies: discoloration of the tongue [15], thrombocytosis [11], and renal damage [11].

**Table 1:** Single-rate meta-analysis of adverse events after linezolid therapy in 758 children from nine studies

Adverse events	Number of events	Proportion, %	95% CI
Total adverse events	205	8.91	1.64%–36.52%
Diarrhea	39	2.24	0.43%–0.81%
Vomiting	16	2.05	0.99%–4.17%
Rash	13	1.72	1.00%–2.93%
Nausea	13	0.96	0.23%–3.91%
Thrombocytopenia	12	0.68	0.05%–8.47%
Hepatic injury	41	0.51	0.03%–9.23%
Loose stools	8	0.30	0.01%–5.74%
Abdominal pain	13	0.23	0.01%–4.75%
Neutropenia	19	0.21	0.00%–9.28%
Anemia	5	0.16	0.00%–5.78%

*CI* confidence interval

**Fig. 2:**
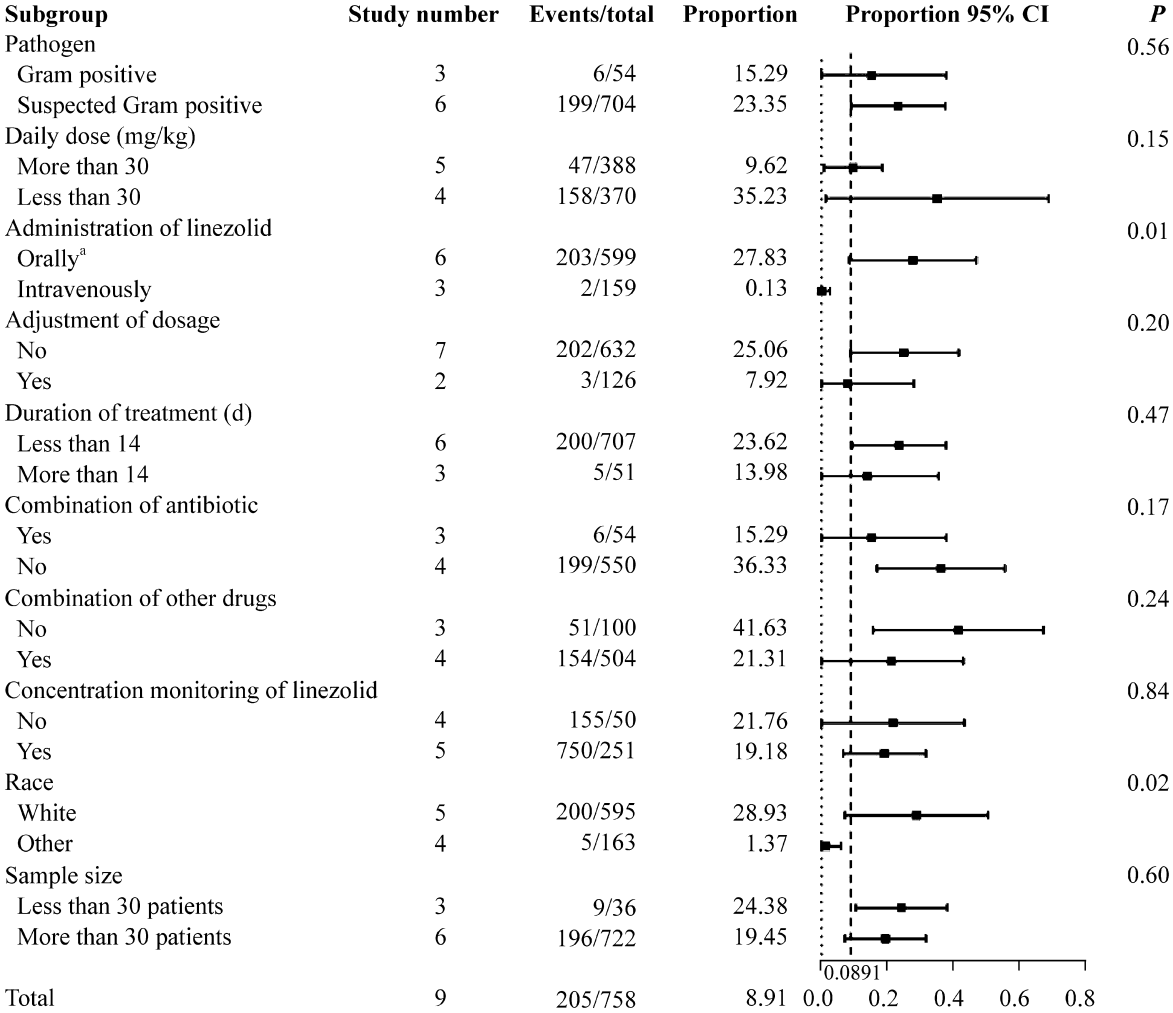
Subgroup analysis of the total adverse events in children after linezolid therapy. *CI* confidence interval. ^a^Including patients receiving sequential therapy with linezolid

**Fig. 3:**
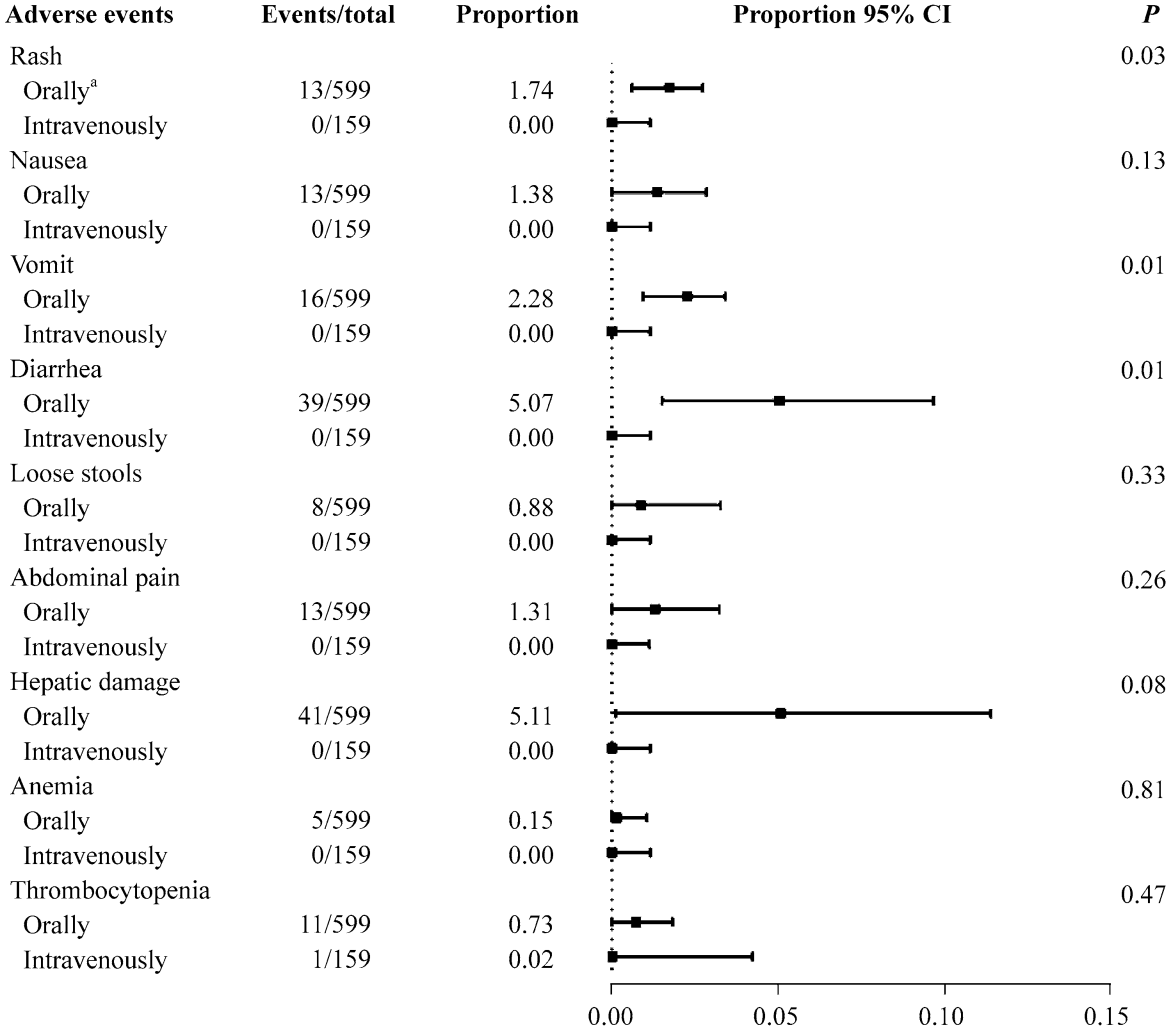
Subgroup analysis of specific adverse events stratified by the route of administration. *CI* confidence interval. ^a^Including patients receiving sequential therapy with linezolid

**Fig. 4:**
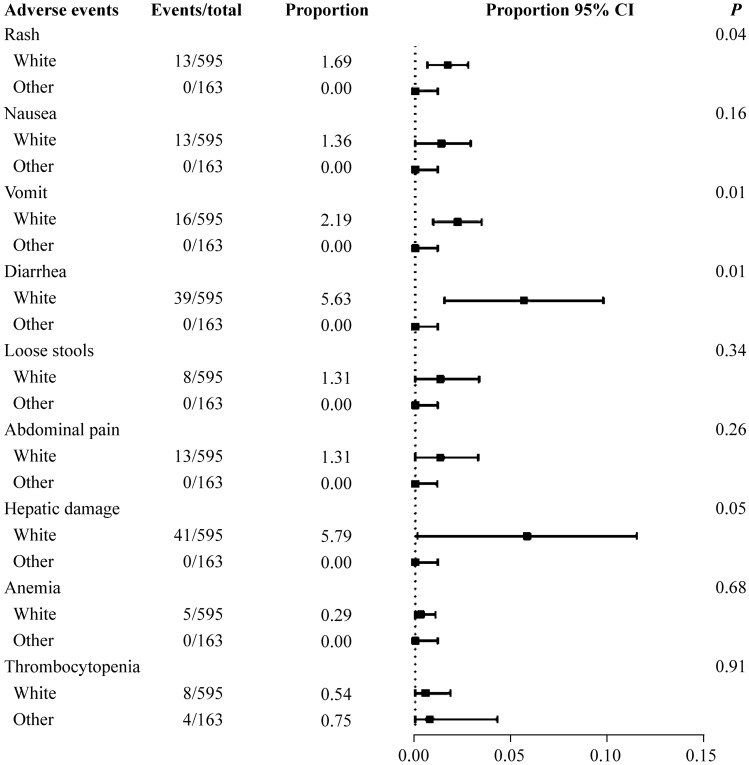
Subgroup analysis of specific adverse events stratified by race. *CI* confidence interval

The subgroup analysis of the total adverse events after linezolid therapy showed a significant difference in the route of linezolid administration and the race of patients (Fig. 2). The proportion was 27.83% in patients who received oral or sequential therapy (95% CI =  8.51%–47.16%). For those who received linezolid via the intravenous route alone, the proportion was 0.13% (95% CI = 0.00%–2.60%); there was a significant difference between the groups (*P* = 0.01). The oral and sequential therapy groups included six studies [8, 10, 11, 14–16], and the intravenous group included three studies [12, 13, 17]. The analysis of specific adverse events stratified by the route of administration showed a significant difference between the subgroups for rash, vomiting, and diarrhea (*P* = 0.03, 0.01 and 0.01, respectively) (Fig. 3). Oral medication seemed to result in more adverse events. In Fig. 4, the studies were stratified by the race of the patients. The white group included seven studies [8, 10–12, 14–16], and the other group included three studies [13, 16, 17]. Significant differences between these subgroups were shown in rash, vomiting, and diarrhea (*P* = 0.04, 0.01 and 0.01, respectively). The following specific adverse events were not significantly different in the subgroups: nausea, loose stools, abdominal pain, hepatic injury, anemia, and thrombocytopenia.

### Efficacy assessed by clinical and microbiological outcomes

**Fig. 5:**
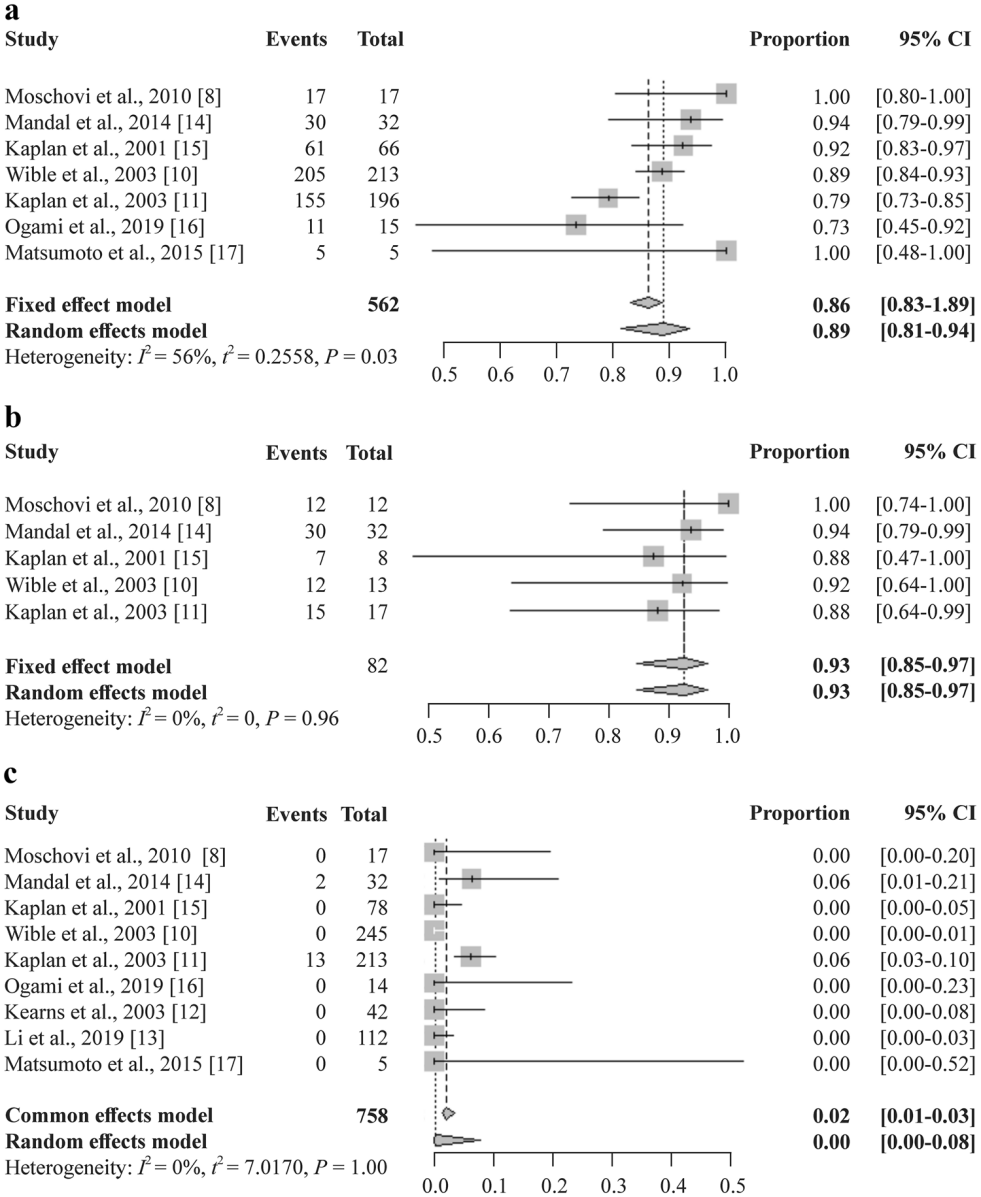
Single-rate meta-analysis of clinical improvement (**a**), pathogen eradication (**b**), and all-cause mortality (**c**) in children after linezolid therapy. *CI* confidence interval

**Fig. 6:**
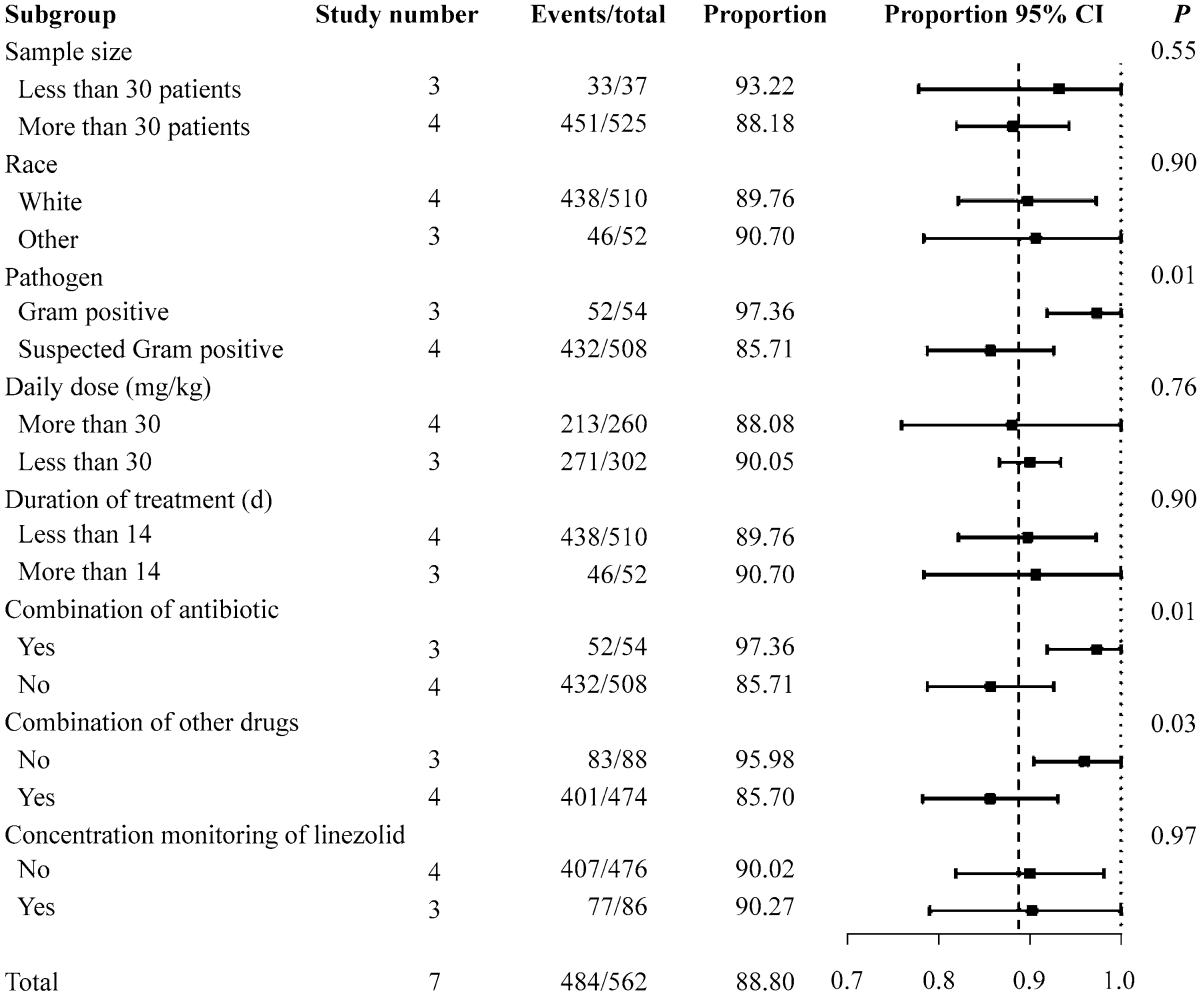
Subgroup analysis of clinical improvement in children after linezolid therapy. *CI* confidence interval

The overall clinical outcomes after linezolid therapy are shown in Fig. 5a [8, 10, 11, 14–17]. A total of 562 people from seven studies were included in the clinical improvement analysis. The effectiveness of treatment was achieved in 88.80% of the patients (95% CI = 81.31%–93.52%, *I*^2^ = 56%, *P* = 0.03). The subgroup analysis of clinical improvement is shown in Fig. 6. The subgroup analysis showed a significant difference with regard to pathogen and combination medication. The clinical improvement was 97.36% in patients diagnosed with specific Gram-positive bacterial infections (95% CI = 91.90%–1.00%) [8, 14, 17]. In patients without bacteriology diagnosis, the proportion was 85.71% (95% CI = 78.78%–92.63%) [10, 11, 15, 16]. There was a significant difference between the two groups (*P* = 0.01). Regardless of the type of drug combinations, the differences between the subgroups were significant.

The overall microbiological outcomes are shown in Fig. 5b [8, 10, 11, 14, 15]. A total of 82 people from five studies were included in the pathogen eradication analysis. The effectiveness of treatment was achieved at 92.68% (95% CI = 84.66%–96.68%, *I*^2^ = 0%, *P* = 0.96). The all-cause mortality outcomes are shown in Fig. 5c [8, 10–17]. A total of 758 patients from nine studies were included. Overall, the proportion was 0.16% (95% CI = 0.00%–7.75%, *I*^2^ = 0%, *P* = 1.00). The subgroup analysis of pathogen eradication (Supplementary Fig. 1) and all-cause mortality (Supplementary Fig. 2) are shown as supplementary data; there was no significant difference between the groups.

## Discussion

A large number of drug-resistant Gram-positive bacterial infections in children have been recognized. Despite its widespread use in clinical practice, there is no consensus on the adverse events or efficacy of linezolid in children. Our study included 758 children from two RCTs and seven prospective noncomparative studies. The overall proportion of adverse events was less than 10%; the events were mostly mild and did not result in the discontinuation of therapy or death, which indicated good tolerance in the children. Oral medication seemed to result in more adverse events, with rash and gastrointestinal reactions being the most common. The overall proportion of clinical improvement and microbial eradication after using linezolid was relatively high (approximately 90% both) among children included in the single rate meta-analysis. Thus, pathogen-based therapy is recommended.

Regarding specific adverse events, diarrhea and vomiting accounted for most of the adverse events. Being mild and reversible, they were not reported to lead to the discontinuation of linezolid therapy but still had a negative impact on the medication experience in children. Linezolid-related bone marrow suppression is also an important adverse event. Interestingly, the incidence of bone marrow suppression in children seems to be lower than that in adults. A study of long-term therapy in adults found that almost half of patients developed reversible thrombocytopenia, and 25% of patients developed anemia, both observed within an average of approximately one month [18]. In our study, thrombocytopenia and anemia were rare, with each having a proportion of less than 1%. Underlying diseases and baseline conditions could affect the severity of illness. Most cases of bone marrow suppression are mild, and severe cases are mainly reported in children with severe underlying disorders [8]. According to a retrospective study in adults, prolonged treatment was a significant risk factor for linezolid-related thrombocytopenia [19]. However, there have been few studies of linezolid therapy lasting longer than 28 days in children.

Although the incidence of rash ranked third among the specific events in our study, it was almost five times lower than that with vancomycin [11]. Studies estimate that 1.6%–14% of children have vancomycin reactions that lead to histamine release, which might have resulted in the discontinuation of the medication [20]. In the current study, linezolid appeared to be safer in terms of rash events. Rash and nephrotoxicity are the main potential toxicities of vancomycin. Renal toxicity has been reported in 12.6%–27.2% of pediatric patients following conventional doses of vancomycin therapy [21]. However, these adverse events are quite rare in the linezolid population. In general, linezolid seems less likely to induce allergic reactions or nephrotoxicity in children. There was no significant difference in gastrointestinal and hematologic events [22].

A study of anti-MRSA agent sales in Japan found that the ratio of oral linezolid to total linezolid rose from 25.5% to 39.9% between 2006 and 2015 [2]. Our study found that oral and sequential therapies might lead to more vomiting and diarrhea events than intravenous therapy. Despite the higher incidence, most adverse events were mild to moderate. In an RCT comparing oral linezolid and cefadroxil for treating uncomplicated SSSIs in children, approximately half of the patients in the linezolid group reported at least one adverse event. Similar to our study, the highest rate of diarrhea was 7.8%, with no myelosuppression after the medication [10]. Because of the more rigorous design of RCTs, they may provide more accurate results than uncontrolled prospective studies and thus might bias the results of this study. However, several studies attributed more adverse events to oral administration in adults, which were dominated by gastrointestinal adverse events [5, 6, 23]. Despite the higher incidence of rash and gastrointestinal adverse reactions, oral linezolid could be considered in children in clinical practice, since the events are mild and acceptable. In addition, we found that race may also affect the proportion of gastrointestinal events and rash.

Regarding efficacy, linezolid is superior to vancomycin. The incidence of drug-resistant Gram-positive bacterial infections, such as MRSA pneumonia, is increasing in children. A study reported that linezolid and vancomycin were top-ranking medications based on recent sales data of anti-MRSA agents [2]. A study to monitor the antibiotic resistance of strains around the world confirmed that the in vitro sensitivity to linezolid was as high as 98.9%–100% in 10,620 Gram-positive strains [24]. For MRSA hospital-acquired pneumonia, intravenous vancomycin and linezolid are preferred drugs according to the updated guidelines from the UK published in 2021 [25]. Vancomycin has a similar antibacterial spectrum compared with linezolid and is commonly used in clinical practice. Linezolid was shown to have slightly better clinical and microbiological efficacy in treating adult MRSA-related infections [22]. However, in children with MRSA, there was no significant difference in clinical or microbiological efficacy between linezolid and vancomycin, both of which were approximately 90% [26]. A review in 2017 showed that the overall rate of VRE colonization was 5% in hospitalized children, who were 8.75 times more likely to develop subsequent VRE infection [27]; the sensitivity study indicated broad prospects for application. With the advantages of tissue penetration and dosage selection, linezolid may have notable potential in clinical practice. However, more research is needed, particularly in terms of the specific pathogen and infection site. Studies have shown that different infection sites and the severity of infections might affect efficacy. For example, the cure rate of adult bloodstream infections was approximately 50%, which is much lower than that for the SSSIs mentioned above [28]. The present study reveals the high overall clinical and microbiological efficacy of linezolid. The targeted use of linezolid after pathogen identification may be an effective way to improve clinical efficacy. This can also explain the difference between the different types of combined medications considered in the current study. Combined antibiotics mainly cover Gram-negative bacteria, and the therapy may be more effective in complicated multiple bacterial infections. In the three studies that identified the etiology, concomitant medication did not lead to a difference in clinical efficacy. Other combined drugs included hormones, chemotherapy drugs, and other drugs for treating underlying diseases, which indicated worse baseline situations.

An advantage of our study is that we eliminated small sample size studies, retrospective studies, and case reports and retained high-quality RCTs and prospective studies, which improved the quality of the article. Routes of administration were not summarized previously, and our study found differences in adverse event rates between oral and sequential therapy vs. intravenous therapy.

This analysis has some limitations. First, there has been no newly reported RCT of linezolid in children in the past 20 years. Thus, we included prospective research to ensure the quality of the analysis, and we combined RCTs and prospective studies in the analysis. However, RCTs had stricter records and better quality control. The results might be biased. Second, there was heterogeneity among the included articles, and the evaluation criteria for adverse events and clinical outcomes differed to varying degrees between the studies. For example, thrombocytopenia was defined in one study as platelets less than 75% of baseline or lower limit of normal after using linezolid and as less than 100 × 10^9^/L or 70% of baseline in other studies. However, insufficient sample size and information in the article made it difficult for us to perform some important subgroup analyses, such as infection type and age. We developed a stratification strategy to compensate for heterogeneity and completed some vital prespecified subgroup analyses, such as route of administration. Third, no difference was found in the subgroup analysis of treatment duration. An analysis of microbiological efficacy was not conducted because of sample-size limitations. Treatment courses longer than 14 days were classified as long course treatment, which may have been suboptimal, since treatment could exceed 28 days in clinical practice. Further research seems necessary in the future.

In summary, linezolid appeared effective and safe for treating infections in children. The adverse events in children seemed to differ from those in adults. The incidence of myelosuppression and neurological events were less common in children than in adults. Oral use might cause more adverse reactions, mainly rash and gastrointestinal events. Regarding efficacy, administering medication after identifying the pathogen is more advantageous. Thus, analyzing the efficacy and adverse events of oral and sequential therapy seems relevant.

## Supplementary Information

Below is the link to the electronic supplementary material.

Supplementary file 1 (PDF 231 KB)

## Data Availability

The data used to carry out this systematic review are available upon request from the corresponding author.
