# Supplementary material for: Safety and clinical efficacy of linezolid in children: a systematic review and meta-analysis
Source: World J Pediatr. 2022 Dec 23;19(2):129–38. doi: 10.1007/s12519-022-00650-1 (PMC9928924; doi:10.1007/s12519-022-00650-1)
Supplement: Supplementary file 1 — (PDF 231 KB) [file 12519_2022_650_MOESM1_ESM.pdf]

Subgroup	Study number	Events/total	Proportion	Proportion 95% CI	*P*
Daily dose (mg/kg)					0.56
More than 30	3	57/61	94.97		
Less than 30	2	19/21	90.94		
Combination of antibiotic					0.29
Yes	2	42/44	96.20		
No	3	34/38	89.88		
Combination of other drugs					0.36
No	2	19/20	97.85		
Yes	3	57/62	92.44		
Total	5	76/82	92.68		

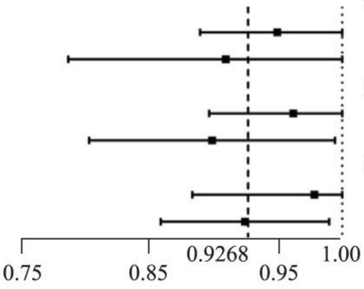

Supplementary Fig. 1 Subgroup meta-analysis of microbial eradication in children after linezolid therapy. *CI* confidence interval

Subgroup	Study number	Events/total	Proportion	Proportion 95% CI	*P*
Pathogen					0.54
Gram positive	3	2/54	2.64		
Suspected Gram positive	6	13/704	0.86		
Daily dose (mg/kg)					0.16
More than 30	5	15/388	2.46		
Less than 30	4	0/370	0.00		
Administration of linezolid					0.24
Orally[a]	6	15/599	1.75		
Intravenously	3	0/159	0.00		
Adjustment of dosage					0.27
No	7	15/632	1.46		
Yes	2	0/126	0.00		
Duration of treatment (d)					0.47
Less than 14	6	13/707	0.84		
More than 14	3	2/51	3.13		
Combination of antibiotic					0.74
Yes	3	2/54	2.64		
No	4	13/550	1.56		
Combination of other drugs					0.19
No	3	0/100	0.00		
Yes	4	15/504	2.86		
Race					0.50
White	5	13/595	1.17		
Other	4	2/163	0.20		
Sample size					0.70
Less than 30 patients	3	0/36	0.00		
More than 30 patients	6	15/722	1.17		
Concentration monitoring of linezolid					0.17
No	4	15/507	2.76		
Yes	5	0/251	0.00		
Total	9	15/758	0.16		

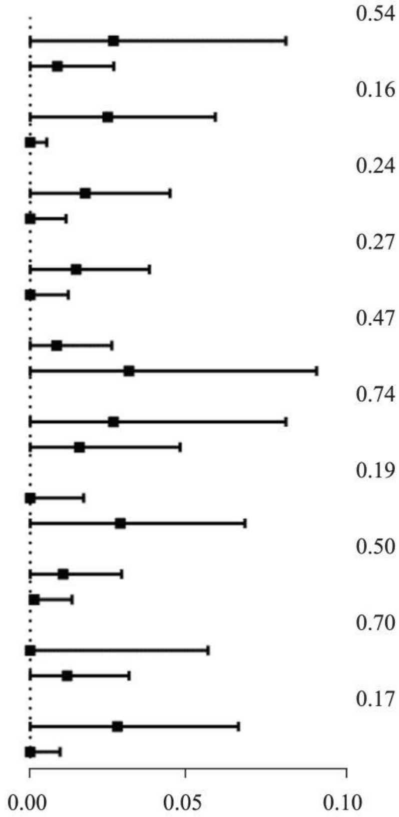

Supplementary Fig. 2 Subgroup analysis of all-cause mortality in children after linezolid therapy. *CI* confidence interval. [a]Including patients receiving sequential therapy of linezolid
